# Pharmacological and Behavioral Effects of the Synthetic Cannabinoid AKB48 in Rats

**DOI:** 10.3389/fnins.2019.01163

**Published:** 2019-10-30

**Authors:** Sabrine Bilel, Micaela Tirri, Raffaella Arfè, Serena Stopponi, Laura Soverchia, Roberto Ciccocioppo, Paolo Frisoni, Sabina Strano-Rossi, Cristina Miliano, Fabio De-Giorgio, Giovanni Serpelloni, Anna Fantinati, Maria Antonietta De Luca, Margherita Neri, Matteo Marti

**Affiliations:** ^1^Department of Morphology, Experimental Medicine and Surgery, Section of Legal Medicine and Laboratory for Technologies of Advanced Therapies (LTTA) Centre, University of Ferrara, Ferrara, Italy; ^2^Section of Legal Medicine, Institute of Public Health, Università Cattolica del Sacro Cuore, Rome, Italy; ^3^Pharmacology Unit, School of Pharmacy, University of Camerino, Camerino, Italy; ^4^Department of Biomedical Sciences, University of Cagliari, Cagliari, Italy; ^5^Department of Psychiatry in the College of Medicine, Drug Policy Institute, University of Florida, Gainesville, FL, United States; ^6^Department of Chemistry and Pharmaceutical Sciences, University of Ferrara, Ferrara, Italy; ^7^Department of Anti-Drug Policies, Presidency of the Council of Ministers, Collaborative Center for the Italian National Early Warning System, Ferrara, Italy

**Keywords:** AKB48, AM251, conditioned place preference (CPP), sensorimotor responses, synthetic cannabinoids, microdialysis, cardiorespiratory changes, prepulse inhibition (PPI)

## Abstract

AKB48 is a designer drug belonging to the indazole synthetic cannabinoids class, illegally sold as herbal blend, incense, or research chemicals for their psychoactive cannabis-like effects. In the present study, we investigated the *in vivo* pharmacological and behavioral effects of AKB48 in male rats and measured the pharmacodynamic effects of AKB48 and simultaneously determined its plasma pharmacokinetic. AKB48 at low doses preferentially stimulated dopamine release in the nucleus accumbens shell (0.25 mg/kg) and impaired visual sensorimotor responses (0.3 mg/kg) without affecting acoustic and tactile reflexes, which are reduced only to the highest dose tested (3 mg/kg). Increasing doses (0.5 mg/kg) of AKB48 impaired place preference and induced hypolocomotion in rats. At the highest dose (3 mg/kg), AKB48 induced hypothermia, analgesia, and catalepsy; inhibited the startle/pre-pulse inhibition test; and caused cardiorespiratory changes characterized by bradycardia and mild bradipnea and SpO2 reduction. All behavioral and neurochemical effects were fully prevented by the selective CB_1_ receptor antagonist/inverse agonist AM251. AKB48 plasma concentrations rose linearly with increasing dose and were correlated with changes in the somatosensory, hypothermic, analgesic, and cataleptic responses in rats. For the first time, this study shows the pharmacological and behavioral effects of AKB48 in rats, correlating them to the plasma levels of the synthetic cannabinoid.

**Chemical Compound Studied in This Article:** AKB48 (PubChem CID: 57404063); AM251 (PubChem CID: 2125).

## Introduction

The New Psychoactive Substances (NPS) comprise a large number of drugs, that are classified based on their chemical structures into different classes such as synthetic cannabinoids, cathinones, opioids, and benzodiazepines, and are widely used because of their easy availability on the Internet (Wood et al., 2014; Orsolini et al., 2015, 2017; Miliano et al., 2016, 2018; Corkery et al., 2017; EMCDDA, 2018). The synthetic cannabinoids (SCBs) are the most popular of the NPS, representing the largest group of substances currently monitored by the EU Early Warning System. Their development on the illicit drug market is likely a response to the popularity of cannabis in many countries. The SCBs mimic the effects of Δ^9^-THC binding in the brain, although they are becoming increasingly chemically different (EMCDDA, 2019).

AKB48 [APINACA, N-(1-adamantyl)-1-pentyl-1H-indazole-3-carboxamide] is a synthetic cannabinoid identified for the first time in 2012 in an herbal mixture in Japan (Uchiyama et al., 2012). It belongs to the third generation of cannabinoids, but it cannot be classified among alkylindoles and cyclohexylphenols (De Luca et al., 2015; Smith et al., 2015; Miliano et al., 2016; De Luca and Fattore, 2018) because of its different chemical structure coming from the increasing demand to synthetize new compounds in order to avoid controls. In particular, AKB48 differs from earlier JWH-type SCBs by having an adamantyl group connected to an indazole moiety through a carboxamide linkage (Canazza et al., 2016).

AKB48 is often added to ready-to-smoke herbal mixtures to enhance psychoactive effects, but as reported recently in forums (e.g., Drugs-forum), a new trend is coming where users buy pure powder and vaporize it to try several synthetic cannabinoids and find out the best one for them. According to users’ opinions, the minimal dose of AKB48 required to vaporize it is about 1 mg, and the onset is gradual until a very pleasant body high, amazing mood, and large smile, until 4 h afterward. On the other hand, the most common adverse effect observed after AKB48 administration is agitation (White, 2017), while other reported side effects are irritability, restlessness, sadness, combativeness, aggression and psychomotor impairments, as reported for SCBs (Brewer and Collins, 2014). Toxicological and forensic analysis revealed AKB48 presence in seized products (Uchiyama et al., 2012; Odoardi et al., 2016) or in biological fluids of people subjected to toxicological control (Karinen et al., 2015; Vikingsson et al., 2015).

From a pharmacodynamics point of view, AKB48 binds in nanomolar concentrations at CB_1_ and CB_2_ human (Uchiyama et al., 2013; Canazza et al., 2016) and mice (Canazza et al., 2016) cannabinoid receptors, suggesting that it could induce similar or higher *in vivo* effects as other SCBs. Preclinical data have reported that AKB48, such as other cannabinoid agonists, induces the typical “tetrad” effect in mice, characterized by hypolocomotion, catalepsy, hypothermia, and acute mechanical and thermal analgesia (Canazza et al., 2016). Moreover, AKB48 causes important alterations of sensorimotor responses (visual, acoustic, and tactile), induces neurological alterations (seizures, hyperreflexia, and myoclonias), and promotes spontaneous aggressive responses in mice by activating CB_1_ receptors (Canazza et al., 2016). It was recently shown that AKB48 induces psychostimulant effects in mice through CB_1_ receptor- and dopamine (DA)-dependent mechanisms. In fact, the motor facilitation induced by AKB48 was prevented by the CB_1_ receptor antagonist AM251, as well as the simultaneous blockade of DA D_1_ and D_2_ receptors (Ossato et al., 2017). Moreover, it was shown that AKB48 and its fluorinated derivative, 5F-AKB48, facilitated extracellular DA release in the nucleus accumbens shell of mice (Canazza et al., 2016; Ossato et al., 2017) and rats (De Luca et al., 2016), suggesting its potential positive involvement in rewarding mechanisms (Gatch and Forster, 2015; Miliano et al., 2016; Ossato et al., 2017), as already established for other synthetic cannabimimetics including JWH-018 (De Luca et al., 2015), JWH-250, and JWH-073 (Ossato et al., 2016).

The metabolism of AKB48 has been identified using a hepatocyte model (Gandhi et al., 2013) and human liver microsomal incubation (Holm et al., 2015). In particular, AKB48 was metabolized in 11 major metabolites, including monohydroxylated, dihydroxylated, trihydroxylated, and mono- and dihydroxylated glucuronide conjugates and dihydroxylated with ketone formation at the N-pentyl side chain (Gandhi et al., 2013).

Despite the presence of these *in vitro* and *in vivo* studies, there is poor preclinical *in vivo* evidence on the addictive properties and pharmaco-toxicological effects of AKB48 in rats. Therefore, the present study aimed to investigate the acute effect of AKB48 on body temperature, acute mechanical and thermal analgesia, motor activity, sensorimotor responses (to visual, acoustic, and tactile stimulation), startle/pre-pulse inhibition tests, conditioned place preference, and modulation of DA release in the mesoaccumbal pathway in adult rats. Also, the effect of AKB48 on cardiorespiratory parameters (hearth rate, breath rate, and SpO2 saturation) was determined. Moreover, to correlate its pharmacological effects with its blood levels, we measured somatosensory responses, body temperature, and mechanical analgesia at timed intervals post-injection, while simultaneously obtaining serial blood specimens for analysis of AKB48 using liquid chromatography tandem mass spectrometry (LC-MS/MS).

## Materials and Methods

### Animals

Male Sprague-Dawley rats (Envigo, Italy) weighing 275–300 g were housed in groups of six per cage, at a constant temperature (22 ± 2°C), humidity (60%), and light/dark cycle (lights on from 08:00 to 20:00 h). Tap water and standard laboratory rodent chow (Mucedola, Settimo Milanese, Italy) were provided *ad libitum* in the home cage. All animal experiments were carried out in accordance with the Guidelines for the Care and Use of Mammals in Neuroscience and Behavioral Research according to Italian (D.L. 116/92 and 152/06) and European Council directives (609/86 and 63/2010) and in compliance with the approved animal policies by the Ethical Committee for Animal Experiments (CESA, University of Cagliari) and the Italian Ministry of Health (Aut. n°162/2016-PR). All animals were handled once daily for 5 min for 5 consecutive d before experimentation began. We made every effort to minimize pain and suffering, and to reduce the number of animals used.

### Drug Preparation and Dose Selection

AKB48, purchased from LGC Standards S.r.l (Milan, Italy), was dissolved in 2% EtOH, 2% Tween 80, and 96% saline and administered intraperitoneally (i.p.; 3.0 mL/kg) at different doses (0.1–3.0 mg/kg i.p.). AM251 (Sigma-Aldrich, Milano, Italy) was dissolved in a solution composed of 10% DMSO, 0.1% Tween 80, and 89.9% distilled water. Drugs were administered 20 min before the beginning of the test. AM251 was administered 20 min before AKB48 injection. The doses of AKB48 and AM251 were selected on the basis of our previous work (Canazza et al., 2016; De Luca et al., 2016).

### Behavioral Studies

The effects of AKB48 were investigated using a battery of behavioral tests widely used in studies of safety pharmacology for the preclinical characterization of NPS in rodents (Ossato et al., 2015, 2018; Vigolo et al., 2015; Canazza et al., 2016; Fantinati et al., 2017; Marti et al., 2019), which in particular have been validated to describe the effects of synthetic cannabinoids in mice and rats (De Luca et al., 2015; Vigolo et al., 2015; Ossato et al., 2015, 2016; Canazza et al., 2016, 2017). To reduce the number of animals used, the behavior of rats was evaluated in four consecutive experimental sections. Moreover, to reduce the stress induced by manipulation, and to confirm the stability and reproducibility over time of the responses of our tests, animals were trained twice per week for 2 weeks before the pharmacological treatment. All experiments were performed between 8:30 a.m. and 2:00 p.m. Experiments were conducted in blind by trained observers working in pairs (Ossato et al., 2016). The behavior of rats (sensorimotor responses) was videotaped and analyzed offline by a different trained operator that gives test scores.

#### Sensorimotor Studies

We studied the voluntary and involuntary sensorimotor responses resulting from different rat reactions to visual, acoustic, and tactile stimuli (Marti et al., 2019).

##### Evaluation of the visual response

Visual response was verified by two behavioral tests that evaluated the ability of the rat to capture visual information even when stationary (the visual object response) or when moving (the visual placing response). The visual object response test was used to evaluate the ability of the rat to see an object approaching from the front or the side, inducing the animal to shift or turn its head or to retreat (modified from Ossato et al., 2015; Marti et al., 2019). For the frontal visual response, a white horizontal bar was moved frontally to the rat’s head; the maneuver was repeated three times. For the lateral visual response, a small dentist’s mirror was moved into the rat’s field of view in a horizontal arc until the stimulus was between the rat’s eyes. The procedure was conducted bilaterally and was repeated three times. The score assigned was a value of 1 if there was a reflection in the rat movement or 0 if not. The total value was calculated by adding the scores obtained in the frontal with that obtained in the lateral visual object response (overall score 9). Evaluation of the visual object response was measured at 0, 10, 30, 60, 120, and 180 min post-injection. The visual placing response test was performed using a tail suspension-modified apparatus able to bring the rat toward the floor at a constant speed of 10 cm/sec (modified from Ossato et al., 2015; Marti et al., 2019). The downward movement of the rat was videotaped. Frame-by-frame analysis allowed us to evaluate the beginning of the reaction of the rat while it was close to the floor. When the rat started the reaction, an electronic ruler evaluated the perpendicular distance in millimeters between the eyes of the rat to the floor. The naïve rats perceived the floor and prepared for contact at a distance of about 27 ± 4.5 mm. Evaluation of the visual placing response was measured at 0, 15, 35, 65, 125, and 190 min post-injection.

##### Evaluation of acoustic responses

Acoustic response measures the reflex of the rat in reply to an acoustic stimulus produced behind the animal. Four acoustic stimuli of different intensities and frequencies were tested (see Marti et al., 2019). Each sound test was repeated three times, giving a value of 1 if there was a response and 0 if not present, for a total score of 3 for each sound. The acoustic total score was calculated by adding scores obtained in the four tests (overall score 12). Evaluation of the visual object response was measured at 0, 10, 30, 60, 120, and 180 min post-injection.

##### Evaluation of tactile responses

Tactile responses were verified through vibrissae, pinna, and corneal reflexes (Marti et al., 2019), and data were expressed as the sum of these parameters. The vibrissae reflex was evaluated by touching the vibrissae (right and left) with a thin hypodermic needle once per side, giving a value of 1 if there was a reflex (turning of the head to the side of touch or vibrissae movement) or 0 if not present (overall score 2). Evaluation of the vibrissae reflex was measured at 0, 10, 30, 60, 120, and 180 min post-injection. The pinna reflex was assessed by touching the pavilions (left and right) with a thin hypodermic needle, the interior pavilions first. This test was repeated twice per side, giving a value of 1 if there was a reflex and 0 if not present (overall score 4). Evaluation of the pinna reflex was measured at 0, 10, 30, 60, 120, and 180 min post-injection. The corneal reflex was assessed by gently touching the cornea with a thin hypodermic needle and evaluating the response, assigning a value of 1 if the rat moved only its head, 2 if it only closed its eyelid, and 3 if it closed its lid and moved its head. The procedure was conducted bilaterally (overall score 6) and was measured at 0, 10, 30, 60, 120, and 180 min post-injection.

#### The “Tetrad” Paradigm for Screening Cannabinoid-Like Effects

##### Evaluation of core and surface body temperature

To better assess the effects of the ligands on thermoregulation, we measured both changes in the core (rectal) and surface (ventral fur) temperature. The core temperature was evaluated by a probe (1 mm diameter) that was gently inserted, after lubrication with liquid Vaseline, into the rectum of the rat (to about 2 cm) and left in position until the stabilization of the temperature (about 10 sec; De Luca et al., 2015; Vigolo et al., 2015). The probe was connected to a Cole Parmer digital thermometer, model 8402. The surface temperature was measured by a Microlife FR 1DZ1 digital infrared thermometer, placed 1 cm from the surface of the abdomen of the rat (Vigolo et al., 2015). Core and surface body temperatures were measured at 0, 15, 35, 65, 125, and 190 min.

##### Evaluation of pain induced by mechanical stimulation of the tail

Acute mechanical nociception was evaluated using the tail pinch tests (modified by Vigolo et al., 2015). A special rigid probe connected to a digital dynamometer (ZP-50N, IMADA, Japan) was gently placed on the tail (in the distal portion), and progressive pressure was applied. When the rat flicked its tail, the pressure was stopped, and the digital instrument saved the maximum peak of weight supported (g/force). A cut-off (500 g/force) was set to avoid tissue damage. The test was repeated three times, and the final value was calculated with the average of three obtained scores. Acute mechanical nociception was measured at 0, 20, 40, 70, 140, and 195 min post-injection.

##### Evaluation of catalepsy in the bar test

In the bar test, the rat’s forelimbs were placed on a bar made of plastic (height 6 cm). The time spent on the bar was measured (immobility cut-off: 20 sec), and akinesia was calculated as the total time spent on the bar after three consecutive trials (total maximal time of catalepsy: 60 s; De Luca et al., 2015; Canazza et al., 2016). The bar test was performed at 0, 20, 40, 70, 140, and 195 min post-injection.

##### Open field test and gross behavior test

An open field, used to measure locomotor activity, consisted of a wooden chamber (45 cm high) with a circular base (75 cm diameter). The floor was divided into 12 sections of similar area by two concentric circles and radial segments. The apparatus was placed in a sound-proof room, illuminated by a white 80 W lamp placed 200 cm over the center of the arena. Rat behavior in the test sessions was videotaped, analyzed, and scored. The following parameters were measured: time spent in the central or peripheral area, locomotor activity, and number of rearing reactions. Each of the behavioral parameters was scored manually by a tally counting method. The behavioral parameters scored were head twitches, wet dog shakes, grooming, licking, number of defecations, and tail rigidity. In the open field test, 21 male Sprague-Dawley rats were used and divided into three groups (*n* = 7 per group) and received different doses of AKB48 according to the CPP protocol. After treatment, the animals were leaved undisturbed for 10 min in the arena and then observed for 30 min.

#### Conditioned Place Preference Paradigm

The CPP chambers consisted of two equally sized compartments interconnected by a guillotine door, adopting a classical conditioning procedure that has been successfully used to assess the rewarding properties of several drugs of abuse. The compartments are differentiated by both visual and tactile cues: the color of the walls in each compartment (white or black) and the texture of the floors (wooden flat or metal wired). The box was placed in a dimly illuminated room. Place conditioning morning sessions ran from 8:30 a.m. to noon, while afternoon sessions ran from 2:30 p.m. to 6 p.m.

##### Experiment 1: effect of AKB48 on conditioned place preference

According to the conditioned place preference paradigm, on day 0 (pre-test), rats freely explored the two compartments for 15 min, and the time spent in each compartment during the exploratory period was measured. Rats that spent 60–70% of the total time on one side were excluded from the experiment. We used an unbiased-like protocol and assigned the drug-paired compartment randomly. On days 1–3 (conditioning phase), male Sprague-Dawley rats (*n* = 24) were divided into three groups (*n* = 8 per group) and given injections of AKB48 (0.1 or 0.5 mg/kg) or vehicle twice daily (9:00 a.m. to 7:00 p.m.) and confined to one compartment for 30 min for 3 days. During these conditioning trials, the animals developed an association between the subjective state produced by the drug and the environmental cues present in the compartment in which they received the drug. On day 4 (test day), rats were allowed to explore the two compartments freely for 15 min, and the time spent in each compartment during the exploratory period was measured.

##### Experiment 2: effect of the CB_1_ antagonist AM251 on AKB48-induced activity on the CPP test

To evaluate if the aversive effect obtained with the higher dose of AKB48 (0.5 mg/kg) was mediated by activation of CB_1_ cannabinoid receptors, male Sprague-Dawley rats (*n* = 32) were divided into four groups (*n* = 8 per group). Group 1 received AKB48 vehicle in both compartments and served as a control. Group 2 was conditioned in one of the two compartments with 0.5 mg/kg of AKB48, and Group 3 was conditioned with AM251 (1 mg/kg). The fourth group received a combination of AM251 and AKB48. CB_1_ antagonist was injected 20 min before the agonist AKB48, which was given 10 min prior to the test.

### *In vivo* Brain Microdialysis Studies

Male Sprague-Dawley rats were anaesthetized with isoflurane gas, and maintained under anaesthesia using a breathing tube under a scavenging system while placed in a stereotaxic apparatus and implanted with vertical dialysis probes (1.5 or 3 mm dialyzing portion for NAc or mPFC, respectively) in the NAc shell (A + 2.2, L + 1.0 from bregma, V-7.8 from dura) or core (A + 1.4; L + 1.6 from bregma; V-7.6 from dura) or in the mPFC (A + 3.7, L + 0.8 from bregma, V-5.0 from dura), according to the rat brain atlas (Paxinos and Watson, 1998).

On the day following surgery, probes were perfused with Ringer’s solution (147 mM NaCl, 4 mM KCl, 2.2 mM CaCl_2_) at a constant rate of 1 μL/min. Dialyzate samples (10 μL) were injected into an HPLC equipped with a reverse-phase column (C8 3.5 μm, Waters, United States) and a coulometric detector (ESA, Coulochem II) to quantify DA. The first electrode of the detector was set at + 130 mV (oxidation), and the second at −175 mV (reduction). The composition of the mobile phase was: 50 mM NaH2PO4, 0.1 mM Na2-EDTA, 0.5 mM n-octyl sodium sulfate, 15% (v/v) methanol, pH 5.5. The sensitivity of the assay for DA was 5 fmol/sample. At the end of the experiment, animals were sacrificed and their brains removed and stored in formalin (8%) for histological examination to verify the correct placement of the microdialysis probe.

### Startle and Pre-pulse Inhibition Analysis

As previously reported (Marti et al., 2019), rats were tested for acoustic startle reactivity in startle chambers (Ugo Basile apparatus, Milan, Italy) consisting of a sound-attenuated, lighted, and ventilated enclosure holding a transparent non-restrictive Perspex^®^ cage (modified version for rats 200 × 90 × 80 mm). A loudspeaker mounted laterally by the holder produced all acoustic stimuli. Peaks and amplitudes of the startle response were detected by a load cell. At the onset of the startling stimulus, 300 ms readings were recorded, and the wave amplitude evoked by the movement of the rat startle response was measured. Acoustic startle test sessions consisted of startle trials (pulse-alone) and pre-pulse trials (pre-pulse + pulse). The pulse-alone trial consisted of a 40 ms 120 dB pulse. Pre-pulse + pulse trial sequences consisted of a 20 ms acoustic pre-pulse, 80 ms delay, and then a 40 ms, 120 dB startle pulse (100 ms onset–onset). There was an average of 15 s (range = from 9 to 21 s) between the trials. Each startle session began with a 10 min acclimation period with a 65 dB broadband white noise that was present continuously throughout the session. The test session contained 40 trials composed by pulse-alone and pre-pulse + pulse trials (with three different pre-pulses of 68, 75, and 85 dB) presented in a pseudorandomized order. Rats were placed in the startle chambers 5 min after treatment with AKB48. The entire startle/PPI test lasted 20 min. The pre-pulse inhibition (PPI) was expressed as the percentage decrease in the amplitude of the startle reactivity caused by the presentation of the pre-pulse. AKB48 (0.1–3 mg/kg i.p.) was administered intraperitoneally, and startle/PPI responses were recorded 15 min (including the 10 min acclimation period) after drug injections.

### Cardiorespiratory Analysis

To monitor cardiorespiratory parameters in awake and freely moving rats with no invasive instruments and minimal handling, a collar with a sensor was applied to continuously detect heart rate, breath rate, and oxygen saturation, with a frequency of 15 Hz (Ossato et al., 2018; Foti et al., 2019). During the experiment, the rat was allowed to freely move in a cage (40 × 40 × 30 cm) with no access to food and water while being monitored by the sensor collar through MouseOx Plus (STARR Life Sciences^®^ Corp., Oakmont, PA, United States) software. In the first hour of acclimation, a fake collar similar to the real one used in the test but with no sensor was used to minimize the potential stress during the experiment. Then, the real collar (with sensor) was replaced, and baseline parameters were monitored for 60 min. Subsequently, AKB48 (0.1–3 mg/kg) or vehicle was administered, and data were recorded for 180 min.

### AKB48 Pharmacokinetic Studies and Behavioral Correlation

To correlate the pharmacological effects of AKB48 with its blood levels, we measured somatosensory responses (visual, acoustic, and tactile), body temperature, and mechanical analgesia in rats at timed intervals post-AKB48 injection (for behavioral tests, see before), while simultaneously obtaining serial blood specimens for analysis of AKB48.

#### Surgical Procedures and Blood Collection

Sixteen male Sprague-Dawley rats were used in the study. Rats were anaesthetized with Equitesin [3 mL/kg intraperitoneal (ip); chloral hydrate 2.1 g, sodium pentobarbital 0.46 g, MgSO 1.06 g, propylene glycol 21.4 mL, ethanol (90%) 5.7 mL, H2O 3 mL] and implanted in the right jugular vein with a catheter, consisting of medical-grade tubing (Silastic, Dow Corning Corporation, MI, United States) according to the technique previously described (Lecca et al., 2006). A stable fixation in the mid-scapular region of the back was embedded by a polypropylene mesh (Evolution, BULEV, weight 48 g/mq, Dipromed, Italy). During the recovery period, at least 7 days after surgery, the catheters were flushed daily with 0.1 mL of gentamicin (40 mg/mL) and with heparinized saline (heparin 250 U/mL in 0.9% sterile saline). Fifteen days after recovery from surgery, rats were trained in handling, behavioral tests, and withdrawing blood from the catheter. Four groups of rats (*n* = 4) randomly received intraperitoneal injection of a single dose of AKB48 (0.1, 0.3, 0.5, or 3.0 mg/kg), and behavioral tests were conducted as previously reported in safety pharmacology studies (see before). Blood specimens (300 μL) were withdrawn via catheters immediately before the behavioral measurements (*T* = 0) and at 20, 40, 70, 140, and 195 min after drug injection. Blood specimens were collected into 1–mL vials containing sodium fluoride (4 mg/mL of blood) as preservative and anticoagulant. After each blood withdrawal, an equal volume of saline solution was infused via the intravenous catheter to maintain volume and osmotic homeostasis.

#### Chemicals

N-(1-adamantyl)-1-pentyl-1H-indazole-3-carboxamide (AKB48) and JWH-209-D9 were purchased from LGC Standards (Milan, Italy). Water, chloroform, formic acid, and methanol were purchased from 3V-Chemicals (Rome, Italy). Ammonium formate was purchased from Agilent (Agilent Technologies, Santa Clara, CA, United States). All reagents and solvents were of LC/MS grade.

#### Sample Preparation

A dispersive liquid-liquid microextraction (DLLME) was performed for sample purification. Three hundred microliter of blood samples were spiked with 10 μL of JWH-209 D9 as an internal standard, achieving the final concentration of 20 ng/mL, and deproteinized with 500 μL of methanol. The sample was centrifuged at 10,000 rpm for 10 min, and 500 μL surnatant was transferred into a 15 mL conical tube containing 1 mL of water, 0.2 g of NaCl, and 100 μL of carbonate buffer, pH 9. In order to obtain the formation of the cloudy solution, 350 μL of a mixture, chloroform/methanol 1:2.5, respectively the extractant and the disperser solvent, was rapidly added to obtain the formation of a turbid mixture. The sample was sonicated for 2 min and then centrifuged at 4000 rpm for 5 min to sediment the fine droplets of the extractant phase at the bottom of the tube. The sediment phase (about 50 ± 5 μL) was transferred into a vial, evaporated under a gentle nitrogen stream, reconstituted in 20 μL of methanol and 80 μL of water with 0.1% formic acid, and 10 μL were injected in the UHPLC-MS/MS system.

#### Instrumental Analysis

The analytical method (UHPLC-MS/MS) and its validation are described in detail elsewhere (Odoardi et al., 2016). N-(1-adamantyl)-1-pentyl-1H-indazole-3-carboxamide (AKB48) and JWH-209-D9 were purchased from LGC Standards (Milan, Italy). Water, chloroform, formic acid, and methanol were purchased from 3V-Chemicals (Rome, Italy). Ammonium formate was purchased from Agilent (Agilent Technologies, Santa Clara, CA, United States). All reagents and solvents were of LC/MS grade. Chromatography was performed using an Agilent 1290 Infinity system, equipped with a binary pump with integrated vacuum degasser, high-performance well-plate autosampler, and thermostatted column compartment modules. The detection system was an Agilent 6460 triple-quadrupole mass spectrometer (Agilent Technologies, Santa Clara, CA, United States) with a Jet-Stream electrospray ionization source. The column was a superficially porous Kinetex C18 column (2.6 μm, 100 × 2.1 mm, Phenomenex, Bologna, Italy). The column temperature was set at 40°C, and the injection volume was 10 μL. The mobile phases used were: (A) 5 mM ammonium formate containing 0.1% formic acid and (B) methanol with 0.1% of formic acid. The mobile phase gradient was from 45% to 100% B within 12 min, plus 3 min of equilibration, for cannabinoids analysis, and from 0 to 90% B within 11 min, plus 3 min of equilibration for stimulants. The flow rate was set to 400 μL/min. The eluate was introduced into the mass spectrometer by means of electrospray ionization (ESI) in the positive mode. The optimized MS parameters were as follows: capillary voltage was set to 4000 V, the ion source was heated up to 350°C, and nitrogen was used as nebulizing and collision gas at 12 L/min and 40 psi, respectively; EM voltage was set to + 1000 V, and nozzle voltage to 2000 V. The detector operated in Multiple Reaction Monitoring (MRM) mode. Transitions selected for AKB48 were 384 → 135, 107, and 93.

#### Behavioral Analysis

To correlate the pharmacological effects of AKB48 with its blood levels, we measured somatosensory responses (visual, acoustic and tactile), body temperature, mechanical analgesia, and catalepsy (as reported before) at each blood withdrawal. Sixteen rats were used in the study. Blood samples were collected immediately after behavioral testing.

### Data and Statistical Analysis

In sensorimotor response experiments, data are expressed in arbitrary units (visual object response, acoustic response, overall tactile response) and percentage of baseline (visual placing response). Core and surface temperature values are expressed as the difference between control temperature (before injection) and temperature following drug administration (Δ°C). Antinociception (tail pinch tests) and catalepsy (bar test) are calculated as percent of maximal possible effect {EMax% = [(test – control latency)/(cut-off time – control)] × 100}. Data are expressed in absolute values [seconds (sec) in time spent in the open field arena, meters (m) for distance traveled, number of head shakes, amount of grooming, number of wet dog shakes, amount of defecation, tail rigidity, and amount of licking]. In microdialysis experiments, data are expressed as percentage of DA basal values. The amount of PPI was calculated as a percentage score for each pre-pulse + pulse trial type:% PPI = 100 - {[(startle response for prepulse + pulse trial)/(startle response for pulse-alone trial)] × 100}. Startle magnitude was calculated as the average response to all pulse-alone trials. Changes in heart rate, breath rate, and SpO_2_ saturation, expressed as heartbeats/minute (bpm), breath rates/minute (brpm), and% oxygen blood saturation, respectively, are expressed as percentage of basal values. Concentration of AKB48 in plasma samples was reported as μg/L.

All the numerical data are given as mean ± SEM. Data were analyzed by utilizing repeated measures ANOVA. Results from treatments showing significant overall changes were subjected to *post hoc* Tukey tests with significance of *p* < 0.05. The statistical analysis of the effects of the individual substances in different concentrations over time and that of antagonism studies in histograms were performed by two- or three-way ANOVA followed by Bonferroni’s test for multiple comparisons. The analysis of the total average effect induced by treatments was performed with one-way ANOVA followed by Tukey’s test for multiple comparisons. Relationships between AKB48 plasma concentrations and behavioral (sensorimotor responses and catalepsy) and physiological (body temperature and mechanical analgesia) changes were assessed using a Pearson’s correlation analysis. The statistical analysis was performed using Prism software (GraphPad Prism, United States).

## Results

### Evaluation of the Visual Object Response

**FIGURE 1:**
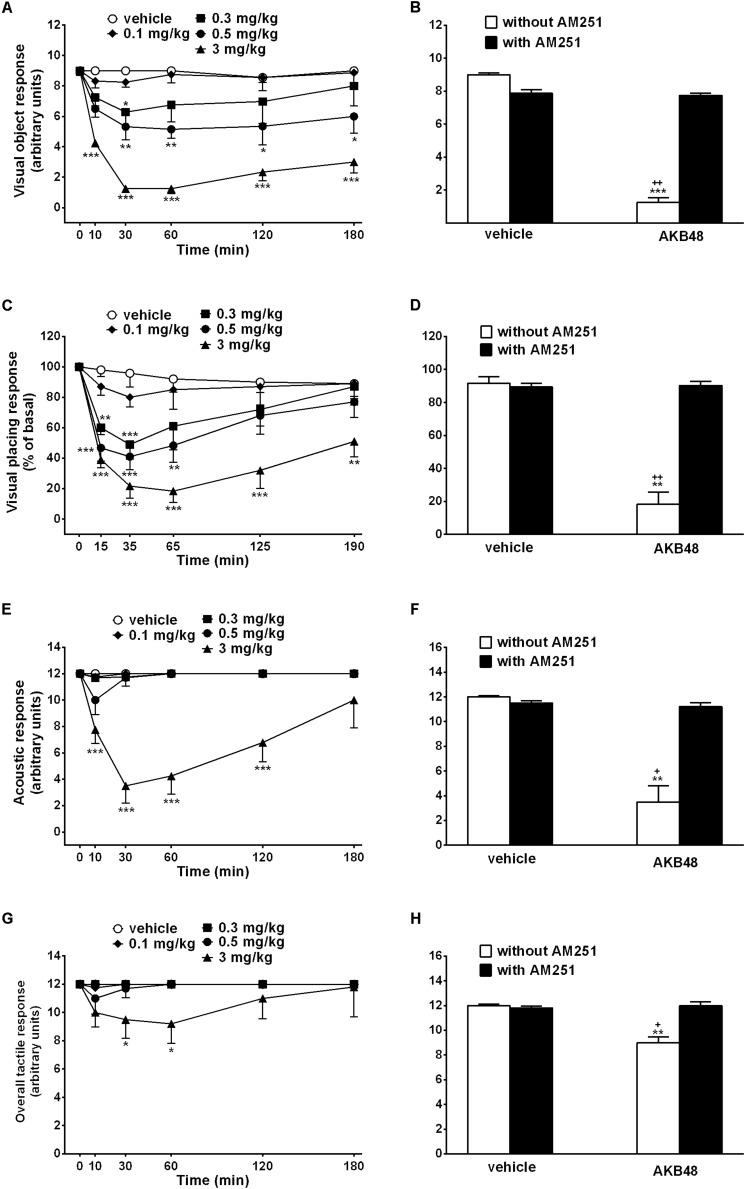
Effect of the systemic administration of AKB48 (0.1–3 mg/kg i.p.) on the visual object **(A)**, the visual placing **(C)**, the acoustic **(E)** and the overall tactile responses in rats. Interaction of AKB48 (3 mg/kg) with the selective CB_1_ receptor antagonist AM251 (1 mg/kg, i.p.) were reported in **B,D,F,H**). Data are expressed (see Materials and Methods) as arbitrary units **(A,B,E–H)** or% of basal **(C,D)** and represent the mean ± SEM of 8 animals for each treatment. Statistical analysis was performed by two-way ANOVA followed by the Bonferroni’s test for multiple comparisons for the dose response curve at different times **(A,C,E,G)** and for the interaction with AM251 **(B,D,F,H)**. ^∗^*p* < 0.05, ^∗∗^*p* < 0.01, ^∗∗∗^*p* < 0.001 vs. vehicle and ^+^*p* < 0.05, **^++^***p* < 0.01 vs. AM251 + AKB48.

Visual object response did not change in vehicle-treated rats over 180 min observation (Figure 1A). Systemic administration of AKB48 (0.1–3.0 mg/kg i.p.) dose-dependently reduced the visual object response in rats. At 0.3 mg/kg, the effect was transient, while the effect caused at the higher doses (0.5 and 3.0 mg/kg i.p.) persisted up to 180 min (Figure 1A); effect of treatment [*F*_(4, 210_ = 59.29, *p* < 0.0001], time [*F*_(5, 210)_ = 11.85, *p* < 0.0001] and time × treatment interaction [*F*_(20, 210)_ = 2.95, *p* < 0.0001]. The inhibition of visual object response induced by the highest dose of AKB48 (3 mg/kg i.p.) was prevented by the pre-treatment with AM251 (1 mg/kg i.p., Figure 1B); effect of treatment [*F*_(1, 28)_ = 15.86, *p* = 0.0004], antagonist [*F*_(1, 28)_ = 4.222, *p* = 0.0493], and interaction [*F*_(1, 28)_ = 14.35, *p* = 0.0007], which alone did not alter the visual object response in rats.

### Evaluation of the Visual Placing Response

Visual placing response slightly decreased in vehicle-treated rats over 190 min observation (∼10% of reduction at 190 min; Figure 1C), and the effect was similar to that observed in naïve untreated animals (data not shown). Systemic administration of AKB48 reduced the visual placing response in rats, and the effect caused at 3 mg/kg i.p. persisted up to 190 min (Figure 1C); effect of treatment [*F*_(4, 210)_ = 31.68, *p* < 0.0001], time [*F*_(5, 210)_ = 15.63, *p* < 0.0001], and time × treatment interaction [*F*_(20, 210)_ = 2.077, *p* = 0.0058]. Visual impairment induced by AKB48 was prevented by the pre-treatment with AM251 (1 mg/kg i.p., Figure 1D): effect of treatment [*F*_(1, 28)_ = 5.234, *p* = 0.0299], antagonist [*F*_(1, 28)_ = 4.671, *p* = 0.0394], and interaction [*F*_(1, 28)_ = 5.663, *p* = 0.0244], which alone did not alter the visual placing response.

### Evaluation of the Acoustic Response

Acoustic response did not change in vehicle-treated rats over 180 min observation (Figure 1E). Systemic administration of AKB48 reduced the acoustic response only at the highest dose, and the effect persisted up to 120 min (Figure 1E); effect of treatment [*F*_(4, 210)_ = 55.63, *p* < 0.0001], time [*F*_(5, 210)_ = 5.59, *p* < 0.0001], and time × treatment interaction [*F*_(20, 210)_ = 5.108, *p* < 0.0001]. The inhibition of acoustic response induced by AKB48 (3 mg/kg i.p.) was prevented by the pre-treatment with AM251 (1 mg/kg i.p., Figure 1F): effect of treatment [*F*_(1, 28)_ = 7.507, *p* = 0.0106], antagonist [F_(1, 28)_ = 3.804, *p* = 0.0612], and interaction [*F*_(1, 28)_ = 5.985, *p* = 0.0210], which alone did not alter the acoustic response in mice (data not shown).

### Evaluation of the Tactile Response

The overall tactile response (vibrissae, corneal, and pinna) did not change in vehicle-treated mice over 180 min observation (Figure 1G). Systemic administration of AKB48 only at the highest dose reduced the tactile responses in rats (∼23% of reduction), and the effect persisted up to 60 min (Figure 1G); effect of treatment [*F*_(4, 210)_ = 5.161, *p* = 0.0006], time [*F*_(5, 210)_ = 0.9673, *p* = 0.4388], and time × treatment interaction [*F*_(20, 210)_ = 0.6782, *p* = 0.8454]. The inhibition of the overall tactile response induced by AKB48 (3 mg/kg i.p.) was prevented by the pre-treatment with AM251 (1 mg/kg i.p., Figure 1H); effect of treatment [*F*_(1, 28)_ = 27.83, *p* < 0.0001], antagonist [*F*_(1, 28)_ = 21.56, *p* < 0.0001], and interaction [*F*_(1, 28)_ = 22.18, *p* < 0.0001], which alone did not alter the acoustic response in mice (data not shown).

### Evaluation of Core and Surface Body Temperature

**FIGURE 2:**
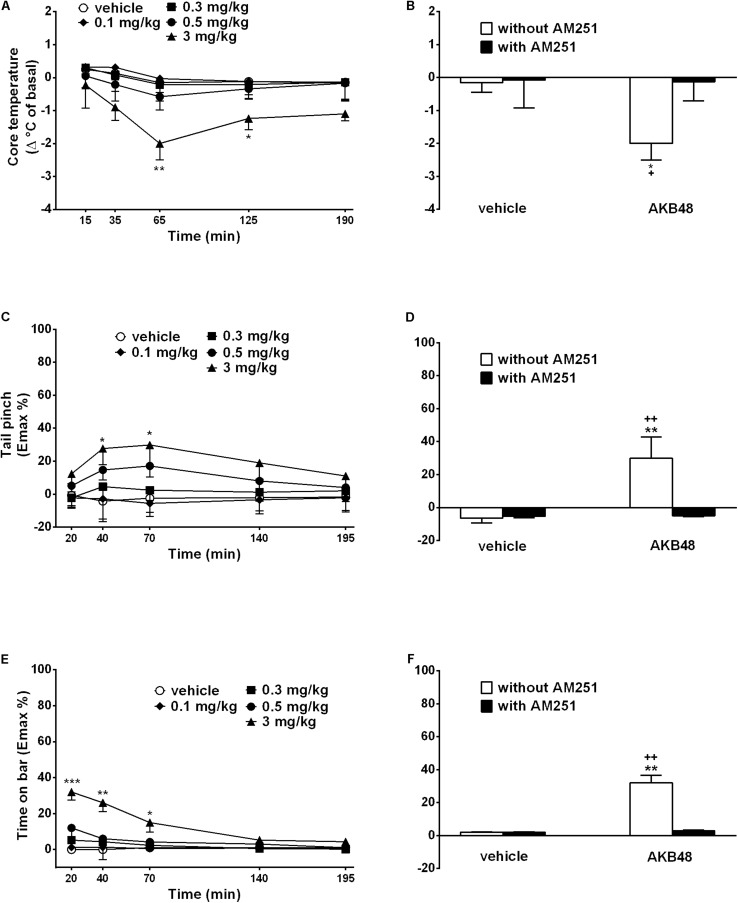
Effect of the systemic administration (0.1–3 mg/kg i.p.) of AKB48 on the rat core temperature **(A)**, on the tail pinch test **(C)** and on the bar test **(E)**. Interaction of AKB48 (3 mg/kg) with the selective CB_1_ receptor antagonist AM251 (1 mg/kg) were reported in **(B,D,F)**. Data are expressed (see section Materials and Methods) as the difference between control temperature (before injection) and temperature following drug administration (Δ°C of basal) and the percentage of maximum effect (Emax %; tail pinch and bar tests) and represent the mean ± SEM of 8 animals for each treatment. Statistical analysis was performed by two-way ANOVA followed by the Bonferroni’s test for multiple comparisons for the dose response curve of each test at different times **(A,C,E)** and for the interaction with AM251 **(B,D,F)**. ^∗^*p* < 0.05, ^∗∗^*p* < 0.01, ^∗∗∗^*p* < 0.001 vs. vehicle and ^+^*p* < 0.05, ^++^*p* < 0.01 vs. AM251 + AKB48.

Body temperature did not change in vehicle-treated rats over 190 min observation. Systemic administration of AKB48 reduced core body temperature only at 3 mg/kg, and the effect was evident only at 65 min (Figure 2A); effect of treatment [*F*_(4, 175)_ = 2.214, *p* = 0.0694], time [*F*_(4, 175)_ = 0.08992, *p* = 0.9855], and time × treatment interaction [*F*_(16, 175)_ = 0.4359, *p* = 0.9711]. The hypothermia induced by AKB48 (3 mg/kg i.p.) was prevented by the pre-treatment with AM251 (1 mg/kg i.p., Figure 2B); effect of treatment [*F*_(1, 28)_ = 13.33, *p* = 0.0011], antagonist [*F*_(1, 28)_ = 15.49, *p* = 0.0005], and interaction [*F*_(1, 28)_ = 14.86, *p* = 0.0006], which alone did not alter the acoustic response. AKB48 did not affect surface body temperature [*F*_(4, 175)_ = 5.129, *p* = 0.0006], time [*F*_(4, 175)_ = 2.757, *p* = 0.0295], and time × treatment interaction [*F*_16, 175)_ = 0.1627, *p* > 0.9999].

### Evaluation of Pain Induced by a Mechanical Stimulus

The threshold to acute mechanical pain stimulus did not change in vehicle-treated rats over 195 min observation (Figure 2C). Systemic administration of AKB48 slightly increased the threshold to acute mechanical pain stimulus in rats in the tail pinch test (Figure 2C); effect of treatment [*F*_(4, 175)_ = 7.266, *p* < 0.0001), time [*F*_(4, 175)_ = 0.6093, *p* = 0.6565], and time × treatment interaction [*F*_(16, 175)_ = 0.2989, *p* = 0.9962]. The effects were prevented by the pre-treatment with AM 251 (1 mg/kg i.p.; Figure 2D); significant effect of treatment [*F*_(1, 28)_ = 4.439, *p* = 0.0442], antagonist [*F*_(1, 28)_ = 2.873, *p* = 0.1012], and interaction [*F*_(1, 28)_ = 3.994, *p* = 0.0554], which alone did not alter the threshold to acute mechanical pain stimuli.

### Evaluation of Catalepsy in the Bar Test

The time spent on the bar did not change in vehicle-treated rats over 195 min observation (Figure 2E). Systemic administration of AKB48 slightly increased the time spent on the bar (Figure 2E); effect of treatment [*F*_(4, 175)_ = 32.19, *p* < 0.0001], time [*F*_(4, 175)_ = 9.843, *p* < 0.0001], and time × treatment interaction [*F*_(16, 175)_ = 3.197, *p* < 0.0001]. The effect was prevented by the pre-treatment with AM 251 (1 mg/kg i.p.; Figure 2F); significant effect of treatment [*F*_(1, 28)_ = 47.09, *p* < 0.0001], antagonist [*F*_(1, 28)_ = 41.21, *p* < 0.0001], and interaction [*F*_(1, 28)_ = 41.21, *p* < 0.0001], which alone did not induce catalepsy in rats.

### Evaluation of Gross Behavior and Spontaneous Locomotion

**FIGURE 3:**
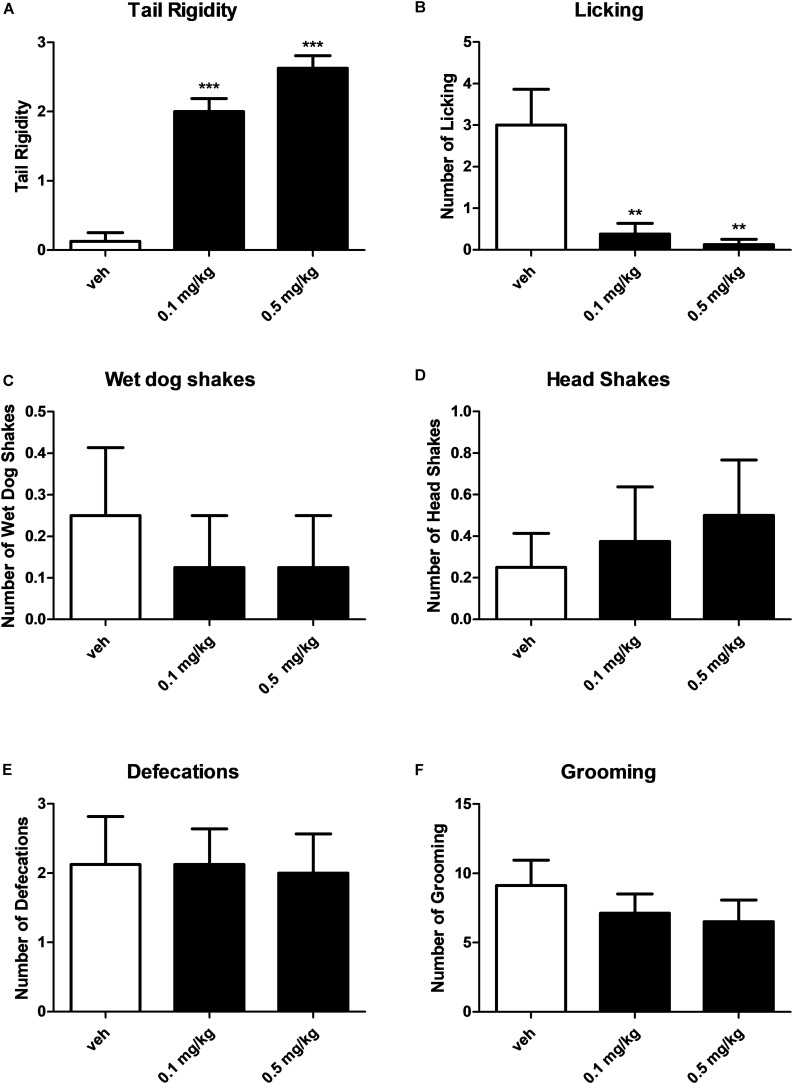
Effect of treatment with AKB48 (0.1 and 0.5 mg/kg, i.p.) on tail rigidity **(A)**, licking **(B)**, wet dog shakes **(C)**, head shakes **(D)**, defecation **(E)**, and grooming behavior **(F)** in rats. Data are expressed (see Materials and Methods) as arbitrary units **(A)** or absolute values **(B–F)** and represent the mean ± SEM of 8 animals for each treatment. Statistical analysis was performed by one-way ANOVA followed by the Bonferroni’s test for multiple comparisons. ^∗∗^*p* < 0.01, ^∗∗∗^*p* < 0.001 vs. vehicle.

**FIGURE 4:**
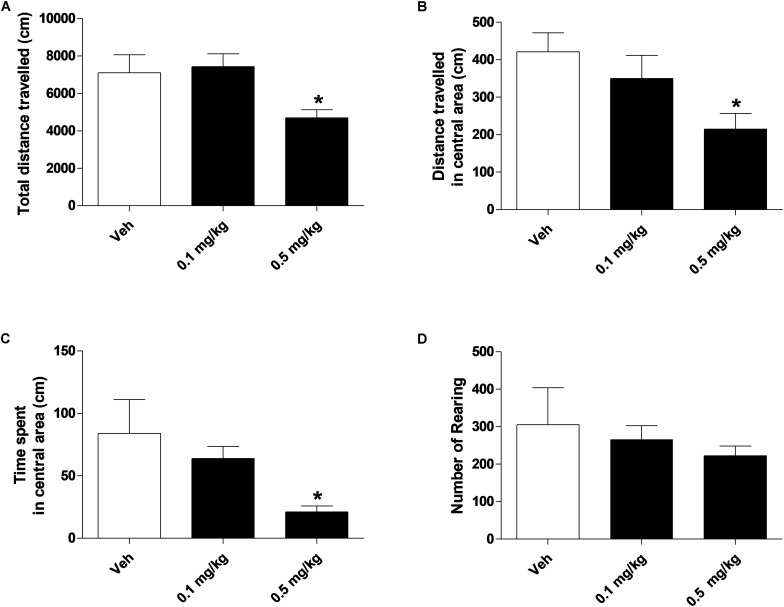
Effect of treatment with AKB48 (0.1 e 0.5 mg/kg, i.p.) on overall total distance traveled **(A)**, total distance traveled and time spent in the central zone **(B,C)** and number of rearings **(D)** in rats. Data are expressed (see section Materials and Methods) as absolute values (cm **A,B**; sec **C**; n° of rearing **D**) and represent the mean ± SEM of 8 animals for each treatment. Statistical analysis was performed by one-way ANOVA followed by the Bonferroni’s test for multiple comparisons. ^∗^*p* < 0.05 vs. vehicle.

Systemic administration with AKB48 (0.1 and 0.5 mg/kg i.p.) induced a significant increase of tail rigidity (Figure 3A) [*F*_(2, 21)_ = 59.9, *p* < 0.0001] and a decrease of licking (Figure 3B) [*F*_(2, 21)_ = 9.12, *p* < 0.001] for both doses tested. However, drug exposure did not affect wet dog shaking (Figure 3C) [*F*_(2, 21)_ = 0.27, *p* > 0.05], head shaking (Figure 3D) [*F*_(2, 21)_ = 0.28, *p* > 0.05], amount of defecation (Figure 3E) [*F*_(2, 21)_ = 0.04, *p* > 0.05], and grooming (Figure 3F) [*F*_(2, 21)_ = 0.72, *p* > 0.05]. Systemic administration with AKB48 (0.1 and 0.5 mg/kg i.p.) reduced spontaneous locomotor activity (Figure 4). In particular, AKB48 at 0.5 mg/kg reduced the distance traveled in the central area (Figure 4A) [*F*_(2, 18)_ = 4.08, *p* < 0.05], the total distance traveled (Figure 4B) [*F*_(2_,_18)_ = 4.31, *p* < 0.05], and the time spent in the central area (Figure 4C) [*F*_(2, 18)_ = 3.63, *p* < 0.05]. On the other hand, treatment with AKB48 had no effect on rearing (Figure 4D) [*F*_(2, 18)_ = 0.44, *p* > 0.05], even if there was a slight decrease.

### Evaluation of the Conditioned Place Preference

**FIGURE 5:**
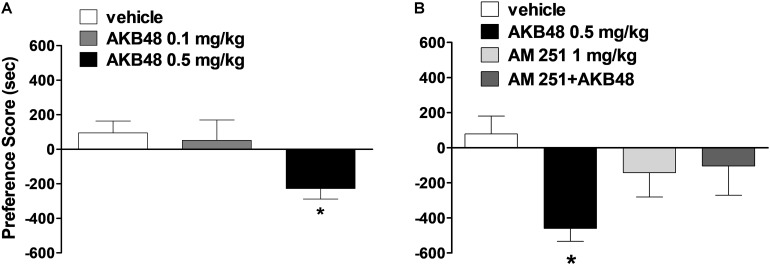
Effect of treatment with AKB48 (0.1 e 0.5 mg/kg, i.p.) on preference place conditioning **(A)**. Interaction of AKB48 (0.5 mg/kg) with the selective CB_1_ receptor antagonist AM 251 (1 mg/kg) on the on preference place conditioning of the rat **(B)**. In each experimental group Δ Time (preference score) was obtained by subtracting the time spent in the drug-paired compartment to that spent in the other compartment. Data are expressed (see section Materials and Methods) as Δ Time and represent the mean ± SEM of 8 animals for each treatment. Statistical analysis was performed by one-way ANOVA followed by the Bonferroni’s test for multiple comparisons. ^∗^*p* < 0.05 vs. vehicle.

Systemic administration with AKB48 at 0.5 mg/kg induced a significant aversive effect on conditioned place preference (Figure 5A) [*F*_(2, 21)_ = 4.13, *p* < 0.05]. The lower dose of 0.1 mg/kg was ineffective. The aversive effect was blocked by administration of AM251 at 1 mg/kg i.p. (Figure 5B) [*F*_(3, 26)_ = 3.22, *p* < 0.05].

### Effect of AKB48 Administration on DA Transmission in the NAc Shell and Core, and in the mPFC of Rats

**FIGURE 6:**
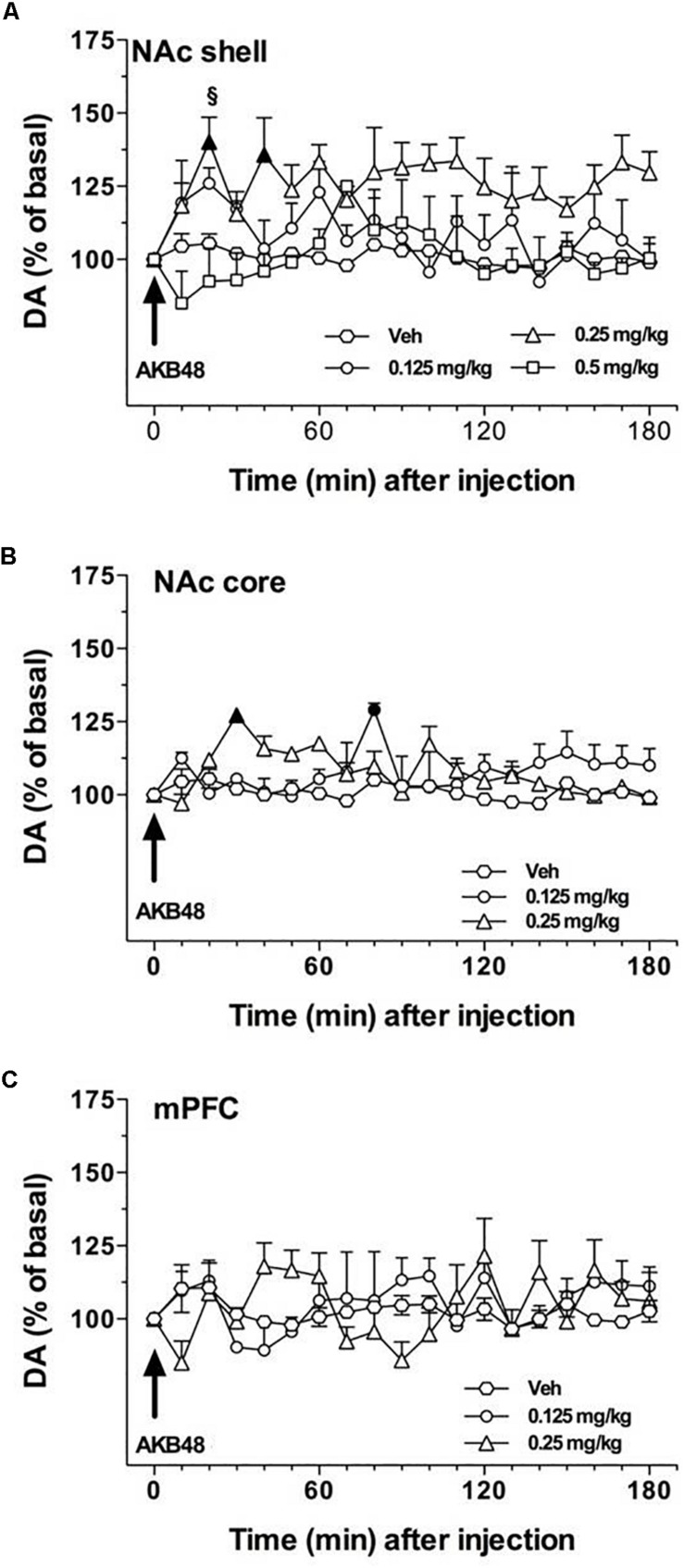
Effect of AKB48 administration on DA transmission in the NAc shell **(A)**, NAc core **(B)**, and mPFC **(C)**. Results are expressed as mean ± SEM of change in DA extracellular levels expressed as the percentage of basal values. The arrow indicates the start of AKB48 i.p. injection at the dose of 0.125 mg/kg (*circles*), 0.25 mg/kg (*triangles*), 0.5 mg/kg (*squares*) or vehicle (*diamonds*) in the NAc shell **(A)**, NAc core **(B)**, and mPFC **(C)**. Statistical analysis was performed by Three-way or two-way ANOVA followed by the Tukey’s HSD *post hoc* test for multiple comparisons. Solid symbol: *p* < 0.05 with respect to basal values; ^§^ *p* < 0.05 vs. NAc core group; ^∗^*p* < 0.05 vs. mPFC group (NAc shell *n* = 11; NAc core *n* = 10; mPFC *n* = 13).

Rat basal values of DA, expressed as fmoles/10 μL sample (mean ± SEM), were: NAc shell 49 ± 5 (*n* = 14), NAc core 48 ± 4 (*n* = 9), and mPFC 14 ± 4 (*n* = 13). In this experiment, we evaluated the effect of three doses of AKB48 on extracellular DA levels in NAc shell (0.125, 0.25, and 0.5 mg/kg i.p.) and two doses in NAc core and mPFC (0.125 and 0.25 mg/kg i.p.). As shown in Figure 6, this synthetic cannabinoid increased DA levels preferentially in the NAc shell (Figure 6A) as compared to the NAc core (Figure 6B) and mPFC (Figure 6C) when administered at 0.25 mg/kg i.p.; lower or higher doses were ineffective in the NAc shell. No significant effects were observed in the NAc core and mPFC. Three-way ANOVA showed a main effect of treatment [*F*_(2, 24)_ = 5.53; ^∗^*p* < 0.05] and time [*F*_(18, 432)_ = 1.651; ^∗^*p* < 0.05]. In animals implanted in the NAc shell, two-way ANOVA showed a main effect of treatment [*F*_(3, 10)_ = 6.126; ^∗^*p* < 0.05]. Tukey’s *post hoc* tests showed a larger increase of dialyzate DA in the NAc shell after 0.25 mg/kg i.p. of AKB48, revealing differences at the 20 and 40 min samples compared to basal values (Figure 6A). In animals implanted in the NAc core, two-way ANOVA showed a main effect of time [*F*_(18, 108)_ = 3.24; ^∗^*p* < 0.0001] and a significant time × treatment interaction [*F*_(36, 108)_ = 3.97; ^∗^*p* < 0.0001]. Tukey’s *post hoc* tests showed a larger increase of dialyzate DA in the NAc core after 0.25 mg/kg i.p. of AKB-48 and after 0.125 mg/kg i.p., revealing differences with respect to basal values (Figure 6B). In animals implanted in mPFC, two-way ANOVA showed no significant effects (Figure 6C).

### Startle/Pre-pulse Inhibition Studies

**FIGURE 7:**
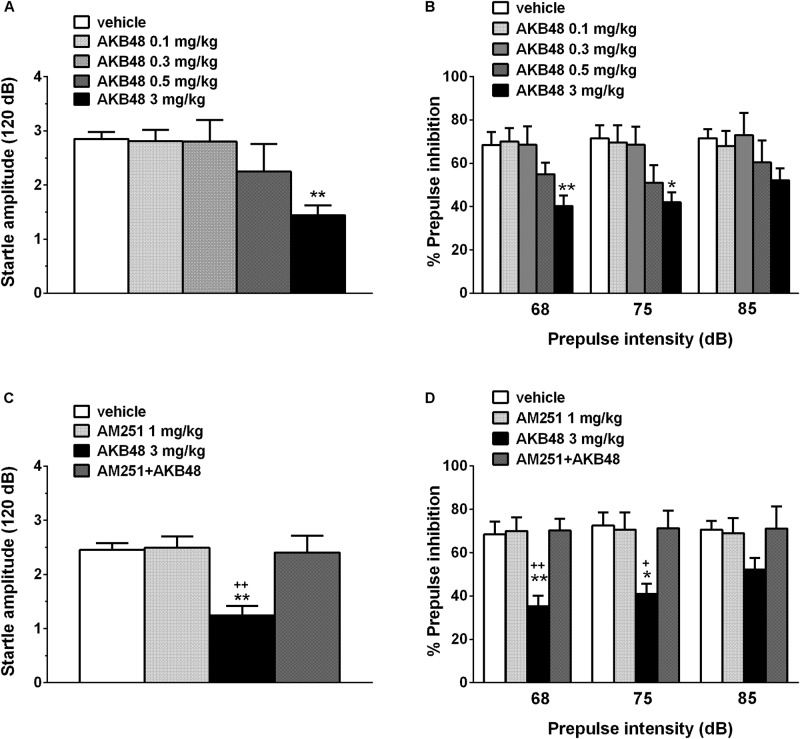
Effect of the systemic administration of AKB48 (0.1–3 mg/kg i.p.) on startle amplitude **(A)** and pre-pulse inhibition (PPI; **B**) in the rat. Effects on PPI are shown for the three prepulse intensities (68, 75, and 85 dB), 15 min after treatment. Effect of AM251 (1 mg/kg i.p.; injected on startle amplitude **(C)** and on AKB48-inhibited PPI **(D)** in the rat was also reported. Data are expressed (see section Materials and Methods) as absolute values (dB; **A,C**) and percentage decrease in the amplitude of the startle reactivity caused by presentation of the pre-pulse (% PPI; **B,D**) and values represent mean ± SEM of 10 animals for each treatment. Statistical analysis was performed by one-way ANOVA followed by Bonferroni’s test for multiple comparisons. ^∗^*p* < 0.05 and ^∗∗^*p* < 0.01 vs. vehicle and ^+^*p* < 0.05, **^++^***p* < 0.01 vs. AM251 + AKB48.

Vehicle injection did not change startle/PPI response in rats, and the effect was similar in naïve untreated animals (data not shown). Administration of AKB48 impaired the startle amplitude in rats (about ∼50% inhibition, Figure 7A) [*F*_(4, 45)_ = 3.579; *P* = 0.0129] at the higher dose tested (3 mg/kg) at 15 min after drug administration. Moreover, AKB48 inhibited the PPI in rats at 68 [*F*_(4, 45)_ = 4.154; *P* = 0.006] and 75 dB [*F*_(4, 45)_ = 3.445; *P* = 0.0154] of pre-pulse intensity (Figure 7B). The inhibitory effect of AKB48 on startle was prevented by AM251 (1 mg/kg i.p.; Figure 7C) [*F*_(3, 36)_ = 7.735; *P* = 0.0004], which alone did not modify the startle response. AM251 also prevented an inhibitory effect of AKB48 on PPI in rats at 68 [*F*_(3, 36)_ = 5.007; *P* = 0.0053] and 75 dB [*F*_(3, 36)_ = 6.837; *P* = 0.0009] of pre-pulse intensity (Figure 7D).

### Cardiorespiratory and Blood Pressure Analysis

**FIGURE 8:**
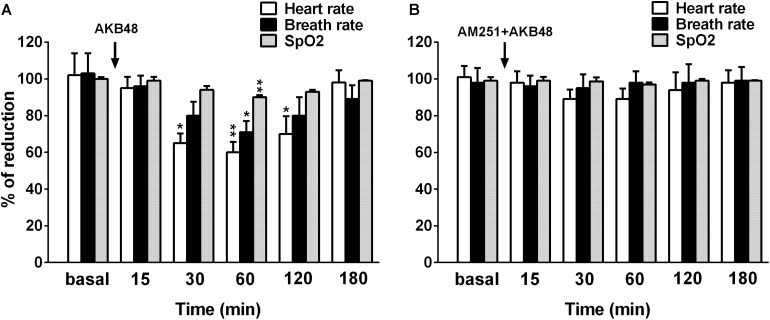
Effect of the systemic administration of AKB48 (3 mg/kg i.p.) on heart rate, breath rate and oxygen arterial saturation in rats **(A)**. Effect of AM251 on AKB48-reduced cardiorespiratory parameters in the rat was reported in **(B)**. For **(A,B)**, data are expressed as the percentage of basal value (heart and breath rate) and as a percentage of oxygen blood saturation (% SpO_2_ saturation) and represent the mean ± SEM of 6 animals for each treatment. Statistical analysis was performed by one-way ANOVA followed by the Bonferroni’s test for multiple comparisons. ^∗^*p* < 0.05, ^∗∗^*p* < 0.01 vs. vehicle.

Systemic administration of AKB48 affected cardiorespiratory parameters in rats (Figure 8). The basal heart rate (430 ± 15 bpm), breath rate (92 ± 8.3 brpm), and SpO_2_ saturation (99.4 ± 1.3%) did not change in vehicle-treated rats over the 3 h observation (Figure 8A). Systemic administration of AKB48 only at the highest dose tested (3.0 mg/kg i.p.) decreased the heart rate of rats (Figure 8A) [*F*_(5, 30)_ = 5.514; *p* = 0.0010]. The effect was significant after 30 min from drug injection (∼36% of reduction); it lasted about 90 min and disappeared at 180 min.

Basal breath rate activity was also reduced by highest dose of AKB48 (Figure 8A) [F_(5, 30)_ = 2.054; *p* = 0.0994]. The effect was significant after 60 min from drug injection (∼30% of reduction) and disappeared at 120 min. Basal SpO_2_ saturation was transiently decreased by the highest dose of AKB48 (Figure 8A) [*F*_(5, 30)_ = 8.227; *p* < 0.0001]. The effect was significant after 60 min from drug injection (∼10% of reduction) and disappeared at 120 min. The CB_1_ receptor antagonist AM251 at 1 mg/kg did not affect the cardiorespiratory parameters and completely prevented the effects of AKB48 at 3 mg/kg (Figure 8B).

### AKB48 Pharmacokinetic Studies and Behavioral Correlation

**FIGURE 9:**
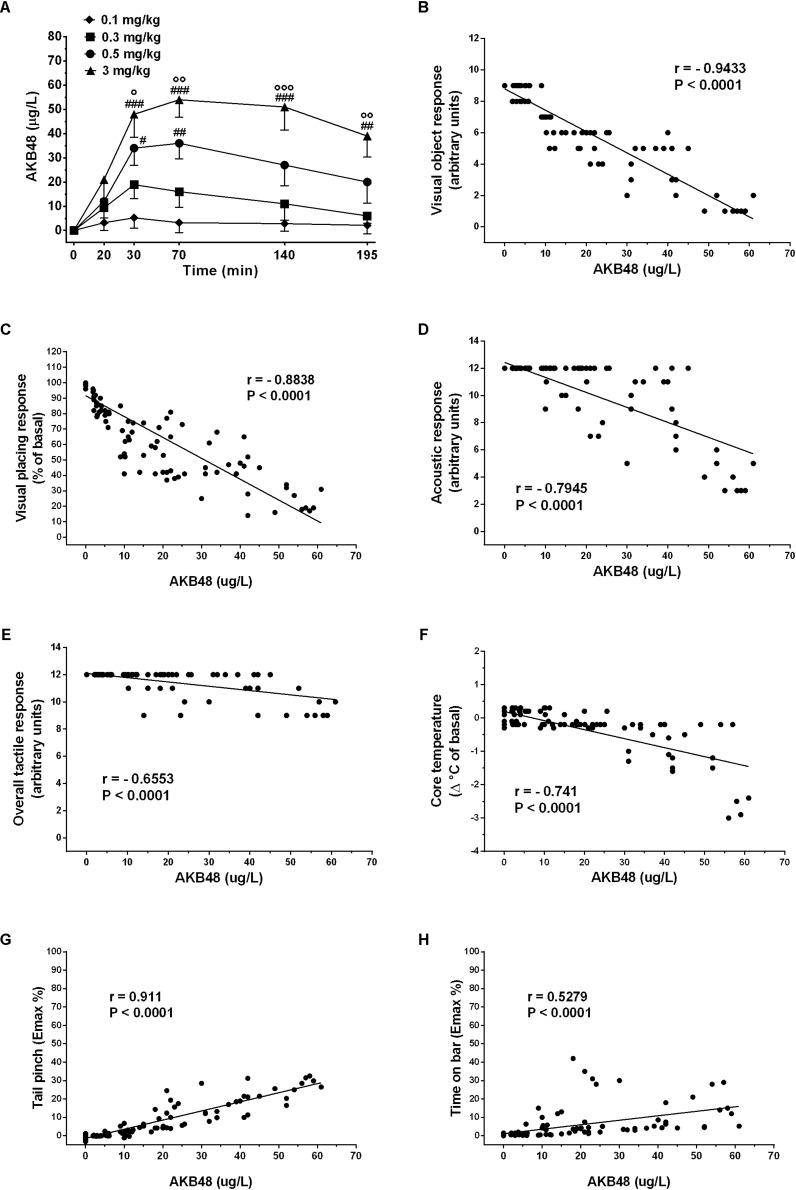
Time-concentration profiles for AKB48 in rats **(A)**. Rats fitted with indwelling jugular catheters received AKB48 doses of 0.1, 0.3, 0.5, or 3.0 mg/kg i.p. at time zero. Blood samples were withdrawn via the catheters at 20, 40, 70, 140, and 195 min after AKB48 injection, and plasma specimens were assayed for analytes using LC-MS/MS. Data are expressed as absolute values (μg/L) and represent the mean ± SEM of 4 rats/group. Statistical analysis was performed by two-way ANOVA followed by the Bonferroni’s test for multiple comparisons for the dose response curve of each test at different times. Correlations between plasma concentrations of AKB48 vs. visual object responses **(B)**, visual placing responses **(C)**, acoustic responses **(D)**, overall tactile responses **(E)**, body core temperatures **(F)**, mechanical analgesia **(G)** and catalepsy **(H)**. Each point in the correlation graphs is plotted by correlating the AKB48 concentrations (μg/L) in blood samples against the behavioral effects observed in rats taken at the same time period. Statistical analysis and correlation were performed by Pearson’s test. Pearson’s *r*- and *P*-values are shown. ^#^*p* < 0.05, ^##^*p* < 0.01, and ^###^*p* < 0.001 vs. AKB48 at 0.1 mg/kg; °*p* < 0.05, °°*p* < 0.01, and °°°*p* < 0.001 vs. AKB48 at 0.3 mg/kg.

AKB48 peak plasma concentration was reached after 30 min at low doses (0.1 and 0.3 mg/kg) and after 60 min at higher doses. Mean values obtained ranged from 4 to 52 ng/mL. After a low decrease following the peak concentration, plasmatic concentrations remained quite stable for the following 120 min. Plasma time-concentration profiles for AKB48 were significantly affected by dose [*F*_(3, 72)_ = 32.5, *p* < 0.0001], time [*F*_(5, 72)_ = 11.74, *p* < 0.0001] and time treatment interaction [*F*_(15, 72)_ = 2.153, *p* = 0.0162], with concentrations rising linearly as dose increased (Figure 9A). AKB48 concentrations after a dose of 0.5 mg/kg were significantly greater than those after 0.1 mg/kg at 40 and 70 min post-injection, whereas concentrations after 3 mg/kg were greater than those after 0.1 and 0.3 mg/kg up to 195 min post-injection. Because we measured pharmacodynamic and pharmacokinetic endpoints from the same rats, we were able to examine relationships between somatosensory responses (visual, acoustic and tactile), body temperature, mechanical analgesia, catalepsy and AKB48 concentrations in plasma. The correlation findings are depicted in Figure 9. Visual object response (Figure 9B; Pearson’s *r* = –0.9433, *P* < 0.0001), visual placing response (Figure 9C; Pearson’s *r* = –0.8838, *P* < 0.0001), acoustic response (Figure 9D; Pearson’s *r* = –0.79.45, *P* < 0.0001), overall tactile response (Figure 9; Pearson’s *r* = –0.6553, *P* < 0.0001), body temperature (Figure 9F; Pearson’s *r* = –0.741, *P* < 0.0001), mechanical analgesia (Figure 9G; Pearson’s *r* = 0.911, *P* < 0.0001), catalepsy (Figure 9H; Pearson’s *r* = 0.5279, *P* < 0.0001) were significantly correlated to AKB48 plasma concentrations.

## Discussion

This is the first study showing through a battery of behavioral tests, the effects caused by the third-generation synthetic cannabinoid AKB48 on “tetrad,” sensorimotor, motor, neurochemical, cardiorespiratory responses, place preference conditioning, and pre-pulse inhibition tests in adult rats. Moreover, AKB48 concentrations in the blood of rats were also monitored and correlated with behavioral measurements.

**FIGURE 10:**
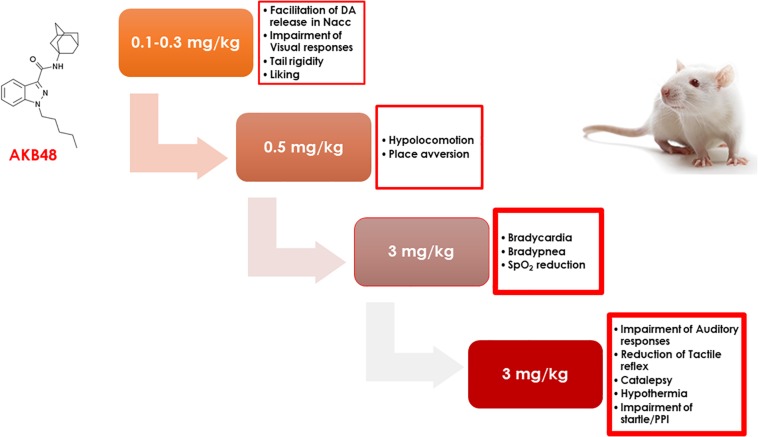
The scheme represents the progressive appearance of pharmacological and behavioral effects in rat as a function of the dose of AKB48 (0.1–3 mg/kg, i.p.) administered.

Consistent with a previous study in mice (Canazza et al., 2016), we showed that the administration of increasing doses of AKB48 causes the progressive onset of different pharmaco-behavioral effects in rats. In particular, AKB48 at low doses (0.1–0.3 mg/kg) mainly inhibits visual sensorimotor responses and preferentially facilitates the release of DA in the NAc shell; increasing the doses of AKB48 (0.5 mg/kg), hypokinesia and place aversion are then observed. At the higher dose (3 mg/kg), cardiorespiratory alterations (bradycardia, bradypnea, and SpO2 reduction), analgesia, hypothermia, reduction of acoustic and tactile sensorimotor responses, and alteration of sensory gating (Figure 10) are observed.

All these behavioral and neurochemical effects were fully dependent on CB_1_ receptor stimulation since they are completely prevented by the administration of the selective CB_1_ receptor antagonist/inverse agonist AM251, as previously reported in the mouse model (Canazza et al., 2016). AKB48 in the range-doses tested (0.1–3.0 mg/kg) reproduced the typical “tetrad” in rats, characterized by hypothermia (at 3 mg/kg), analgesia (at 3 mg/kg), catalepsy (at 3 mg/kg), and hypolocomotion (at 0.5 mg/kg). These findings are in line with previous studies showing the effectiveness of different SCBs based on indole and indazole scaffolds in inducing the overall “tetrad” effect (De Luca et al., 2015), hypothermia, and catalepsy (Carlier et al., 2018; Elmore and Baumann, 2018) or hypothermia (Banister et al., 2015a, b, 2016; Schindler et al., 2017) in rats.

AKB48 is less active than JWH-018 in inducing the “tetrad” effect in the rat (De Luca et al., 2015). From a chemical structural point of view, AKB48 differs from naphthoylindole SCBs (JWH-type) by having an adamantyl group connected to an indazole moiety through a carboxamide linkage (Uchiyama et al., 2012). The presence of the adamantil group rather than the indazolic structure could cause its lower efficacy and potency *in vivo*. In fact, indazole synthetic cannabinoids 5F-AMB, MDMB-FUBINACA (Banister et al., 2016), AB-FUBINACA, and AB-PINACA (Banister et al., 2015a) cause hypothermia in the rat in a range of concentrations similar to those of indolic compounds such as JWH-018 and AM-2201 (Banister et al., 2015b). It appears evident that the presence of an adamantyl group linked to the main structure of the SCBs causes a loss of power and duration of action of the SCBs on the hypothermic effect (Banister et al., 2015b).

Besides this hypothesis, it is also possible that the lower responses could be related to the biotransformation of AKB48, as well as others SCBs, into glucuronitated or monohydroxylated metabolites that can act as neutral antagonists at CB_1_ receptors, dampening the overall activity of the parent drug (Brents et al., 2012; Seely et al., 2012).

Unlike previous studies demonstrating that the analgesic effect on mechanical pain stimuli provoked by JWH-type compounds precede or match the motor impairment (De Luca et al., 2015; Vigolo et al., 2015; Ossato et al., 2016; Canazza et al., 2017), AKB48 firstly induces hypolocomotion (0.5 mg/kg i.p.) and then starts to become an analgesic (3 mg/kg i.p.). This responsiveness is in line with previous studies in mice (Canazza et al., 2016) and is in agreement with evidence showing that small modifications of the molecular structure of SCBs induce consistent disparities among potencies and efficacies of *in vivo* effects (Wiley et al., 1998, 2014; Ossato et al., 2016). In our experimental conditions, the possibility that the acute analgesic effect induced by AKB48 and/or its metabolites (Gandhi et al., 2013; Holm et al., 2015) is due to the activation of peripheral CB_2_ receptors (Guindon and Hohmann, 2008) and should be ruled out since their analgesic effects are fully prevented by the administration of the selective CB_1_ receptor antagonist/inverse agonist AM251. As reported by others (Ossato et al., 2015, 2016; Canazza et al., 2016, 2017) AKB48 at lower doses (0.3 mg/kg) greatly impairs visual sensorimotor responses in rats and reduces acoustic and tactile reflexes at a dose 10 times higher (3 mg/kg). Reig and Silberberg have demonstrated in their recent study that visual information in rodents is elaborated in a subpopulation of neurons selectively localized in the dorsomedial striatum (Reig and Silberberg, 2014), in which CB_1_ receptors are expressed (Tsou et al., 1998; Marsicano and Lutz, 1999). Even though in our study we cannot reveal which brain areas and neural mechanisms are involved in the reduction of visual response of the rat, it is possible to hypothesize that AKB48 could stimulate CB_1_ receptors expressed in thalamocortical-striatal visual circuitry (Tsou et al., 1998; Marsicano and Lutz, 1999; Dasilva et al., 2012; Yoneda et al., 2013), and cause an impairment of the visual function in rats.

It is interesting to note that AKB48 impairs visual sensorimotor responses in rats at a low dose (0.3 mg/kg) that does not cause hypolocomotion (open field studies). These findings reveal that effects induced by AKB48 on visual sensorimotor responses and motor activity are mediated by separate processes and suggest that a decrease in sensory responsiveness does not reflect a disruption of motor function (Ossato et al., 2015).

Our study also demonstrates that AKB48 impairs the acoustic startle response in rats through selective stimulation of CB_1_ receptors. This interpretation is in accordance with previous studies that have proved the effectiveness of acute administration of Δ^9^-THC (Malone and Taylor, 2006; Nagai et al., 2006; Ossato et al., 2015), CP 55940 (Mansbach et al., 1996; Martin et al., 2003), WIN-55,212-2 (Bortolato et al., 2005), JWH-018 (Ossato et al., 2015), JWH-250, and JWH-073 (Ossato et al., 2016) in reducing the acoustic startle reflex in rodents. A recent study on acoustic startle reflex showed that this mechanism is induced by the activation of three serially connected structures that involve the activation of the dorsal cochlear nucleus (Gomez-Nieto et al., 2014). In addition, a study of Tzounopoulos and colleagues showed that CB_1_ receptors are expressed on the presynaptic terminals of parallel fibers in the dorsal cochlear nucleus (Tzounopoulos et al., 2007). Thus it is possible to speculate that AKB48 could impair the acoustic startle reflex in rats by stimulating CB_1_ receptors located in the dorsal cochlear nucleus.

A common feature of cannabinoid drugs is to induce in both humans (Kedzior and Martin-Iverson, 2006) and animals (Peres et al., 2016) a reduction in PPI of the acoustic startle reflex, which is considered an operational measure of the sensory gating (or filtering) that is severely impaired in schizophrenia patients (Javitt and Zukin, 1991). Our study demonstrates that AKB48 impairs the PPI response in rats by the selective stimulation of CB1 receptors. This finding is in agreement with previous studies that have demonstrated the effectiveness of acute administration of Δ9-THC (Malone and Taylor, 2006; Nagai et al., 2006), CP 55940 (Mansbach et al., 1996; Martin et al., 2003), and WIN 55,212-2 (Schneider and Koch, 2002; Wegener et al., 2008) in reducing PPI in rodents. These findings extend to these synthetic compounds the ability to induce information processing deficits and sensory disturbances that may account for their psychotic effects in humans (Every-Palmer, 2010; Every-Palmer, 2011). The acute administration of AKB48 (0.5 mg/kg ip) reduced the locomotor activity and induced aversion in the place preference task. According to our results, place conditioning tests in rodents showed that high doses of Δ9-THC or synthetic cannabinoid agonists such as WIN 55212-2 and HU210 caused a significant place aversion (CPA), counteracted by the CB1 receptor antagonist/inverse agonist SR 141716A (Chaperon et al., 1998; Cheer et al., 2000; Valjent and Maldonado, 2000; Tzschentke, 2007). Similarly, in our test, the aversive effect of high doses of AKB48 was abolished by treatment with the selective CB1 antagonist AM251.

The observation of aversive effects in the CPP paradigm is in line with the inability of AKB48, when administered at the dose of 0.5 mg/kg ip, to increase DA transmission in the NAc shell thus confirming a lack of rewarding properties of high doses of AKB48 analogs (De Luca et al., 2015). However, as demonstrated in this study, an acute administration of 0.25 mg/kg, but not 0.125 mg/kg, affected DA signaling in the NAc shell but not in the NAc core nor mPFC. It is well-established that a selective increase of extracellular DA in the NAc shell, estimated by *in vivo* brain microdialysis in rodents, is a common feature of drugs abused by humans (Di Chiara and Imperato, 1988), including cannabinoids (Tanda et al., 1997; Sidhpura and Parsons, 2011). Therefore, a complete pharmacological and toxicological characterization of NPS generally comprises the measurement of DA levels in specific brain areas (i.e., NAc shell/core/mPFC). For this reason, although already performed in mice (Canazza et al., 2016) and demonstrated for different generations of synthetic cannabinoids (De Luca et al., 2015, 2016), the present study displays a set of microdialysis experiments after the administration of AKB48. We observed that AKB48 selectively stimulated NAc shell DA at the same dose of JWH-018 (De Luca et al., 2015), the prototypical compound of the first generation of SCBs. However, the chemical hindrance of the adamantyl group (Figure 10) may be the reason for a lower (max increase of about 40%, at 20 and 40 min) and less-lasting increase of DA with respect to values observed after the same dose of JWH-018 (0.25 mg/kg ip) (max increase of about 60%, from 20 to 70 min) (De Luca et al., 2015; Miliano et al., 2016).

The present study discloses the possible rewarding properties of AKB48, as well as the peculiar pharmacological profile of SCBs. Indeed, in the response of NAc shell DA, we observed an inverted U-shaped dose-response curve at a dose enclosed between an extremely narrow range of doses (0.125–0.5 mg/kg ip). Nethertheless, few and contrasting studies specifically investigated the relationship between cannabinoid effects in CPP and dopaminergic release in specific rewarding areas (Polissidis et al., 2009; Tampus et al., 2015). Polissidis et al. (2009) demonstrated that a stimulatory low dose (0.1 mg/kg) of the cannabinoid CB1 receptor agonist WIN 55,212-2 on motor activity was not accompanied by place preference, but enhanced dopaminergic activity in the nucleus accumbens shell of rats. Ossato et al. (2017) also demonstrated that the transitory (15 min) psychostimulant effect of AKB48 in mice, facilitating spontaneous locomotion, is mediated by activation of both CB1 and D1/5 and D2/3 dopaminergic receptors, resulting in an increased NAc DA release. In addition, as regards to the synthetic cannabinoid JWH-018, high doses (1 and 3 mg/kg) that induced CB1 receptor-dependent behavioral effects in rats (such as catalepsy and hypomotility) have no effect on DA release in the NAc shell (De Luca et al., 2015), but induced place aversion (Hyatt and Fantegrossi, 2014). Interestingly, only the 0.25 mg/kg dose increased DA release in the NAc shell. On the other side, a lower (0.125 mg/kg) and higher (0.50 mg/kg) doses were ineffective (De Luca et al., 2015). Similarly to JWH-018, the doses of AKB48 that evoke an increase in dopamine signaling are lower than those that induced, for example, hypolocomotion or aversive effects. Taking this into consideration, it could be speculated that low doses of AKB48 exhibit conditioned rewarding effects, that, for higher doses seems to be masked by the prevalent aversive and anxiolytic-like properties. In line with this reasoning, the increased tail rigidity and the reduction of amount of licking could be interpreted as an index of anxiety and related-behaviors. In fact, some of the most established indicators of negative emotional behavior (fear response or anxiety) in the open field test are low ambulation and increased tail rigidity (Sestakova et al., 2013).

The present study shows for the first time that AKB48, at the highest dose tested (3 mg/kg), induces bradycardia in rats through CB1 receptor activation. This action is consistent with previous studies showing the cardiovascular depressive effects induced by different SCBs in rats (Banister et al., 2015a, b, 2016) and is possibly due to the mechanism of sympathoinhibition and enhancement of cardiac vagal tone mediated by CB1-receptors (Schmid et al., 2003). Cardiac alterations, such as palpitations, chest pain, bradycardia, tachycardia, arrhythmias, hypotension, syncope, and ECG changes like T-wave inversion, represent one of the main adverse effects associated with SC use in humans (Hermanns-Clausen et al., 2016; McIlroy et al., 2016; Von Der Haar et al., 2016; Sud et al., 2018). Some literature also describes cases of death by myocardial infarction or cardiac arrest directly attributed to synthetic cannabinoid use (Mir et al., 2011; Ibrahim et al., 2014). Further studies will be carried out to verify if the administration of SCBs can cause direct damage to heart tissue.

In addition to bradycardia, AKB48 induces a CB1 receptor-mediated respiratory depression, as shown by a decrease in respiratory rate (inhibition of about 40% of basal breath rate values) and hypoxia (reduction of about 15% of basal SpO2 values). Recent data suggest that SCBs induced acute respiratory depression (Alon and Saint-Fleur, 2017). This effect was most likely not a consequence of cardiovascular depression, since cardiovascular depression usually leads to a stimulation of the central ventilatory drive, and consequently, to an increase in respiratory frequency (Schmid et al., 2003). The cannabinoids inhibited respiration probably by acting directly in the central nervous system (Pfitzer et al., 2004). However, an additional effect at the periphery cannot be ruled out since cannabinoids could play an important role in the functioning of different peripheral receptors involved in respiratory regulation (e.g., pulmonary stretch receptors, chemo- and baroreceptors). They could also have a direct action on the bronchi altering the airway resistance (Calignano et al., 2000).

Finally, the present study firstly reported a plasma pharmacokinetic profile for AKB48 in rats monitored for about 3 h and correlating plasma levels of AKB48 with different behavioral measurements. As shown in Figure 9, a correlation was observed between plasma concentration and sensorimotor (visual, acoustic and tactile) responses, mechanical analgesia, core temperature and catalepsy. This preclinical study is aimed to extend pharmaco-toxicolgical features of AKB48 in the animal model, however the results obtained could not be correlated with clinical studies due to limited number of samples and information reported in cases of intoxication with this SCB in human.

## Conclusion

The present study discloses, for the first time, the overall progressive pharmacological and behavioral effects induced by the progressive administration of adamantylindazole AKB48 in rats (Figure 10), highlighting its ability to primarily disrupt visual sensimotor responses and facilitate DA release in the NAc shell. With increasing doses, hypokinesia and place aversion were registered. Finally, at higher doses, a reduction of cardiorespiratory signs (bradycardia, bradypnea, and spO2); acoustic and tactile sensimotor responses; and analgesia, hypothermia, and catalepsy were observed.

## Data Availability Statement

The datasets generated for this study are available on request to the corresponding author.

## Ethics Statement

The animal study was reviewed and approved by the Ethical Committee for Animal Experiments (CESA, University of Cagliari) and the Italian Ministry of Health (Aut. n°162/2016-PR).

## Author Contributions

MM, MD, MN, and FD-G contributed conception and design of the study. SB, MT, RA, SS, LS, SS-R, and AF performed experimental sections. SB, SS, and CM organized the database. MT, RA, and CM performed the statistical analysis. MM wrote the first draft of the manuscript. SB, SS, LS, RC, PF, SS-R, CM, GS, MN, and MD wrote sections of the manuscript. All authors contributed to manuscript revision, read and approved the submitted version.

## Conflict of Interest

The authors declare that the research was conducted in the absence of any commercial or financial relationships that could be construed as a potential conflict of interest.

The handling editor declared a past co-authorship with one of the authors, MM.

## Abbreviations

AM251: 1-(2,4-dichlorophenyl)-5-(4-iodophenyl)-4-methyl-N-(piperidin-1-yl)-1H-pyrazole-3-carboxami de
AKB48: N-(1-adamantyl)-1-pentyl-1H-indazole-3-carboxamide
CPP: conditioned place preference
DA: dopamine
NAc: shell Nucleus Accumbens shell
NAc: core Nucleus Accumbens core
mPFC: medial prefrontal cortex
PPI: Prepulse Inhibition
JWH-018: Naphthalen-1-yl-(1-pentylindol-3-yl)methanone.
